# Supplementary material for: Prognostic and Immunotherapeutic Roles of KRAS in Pan-Cancer
Source: Cells. 2022 Apr 22;11(9):1427. doi: 10.3390/cells11091427 (PMC9105487; doi:10.3390/cells11091427)
Supplement: Supplementary file 1 [file cells-11-01427-s001.zip › cells-1666153-supplementary/Supplementary Figure S1.pptx]

## Slide 1
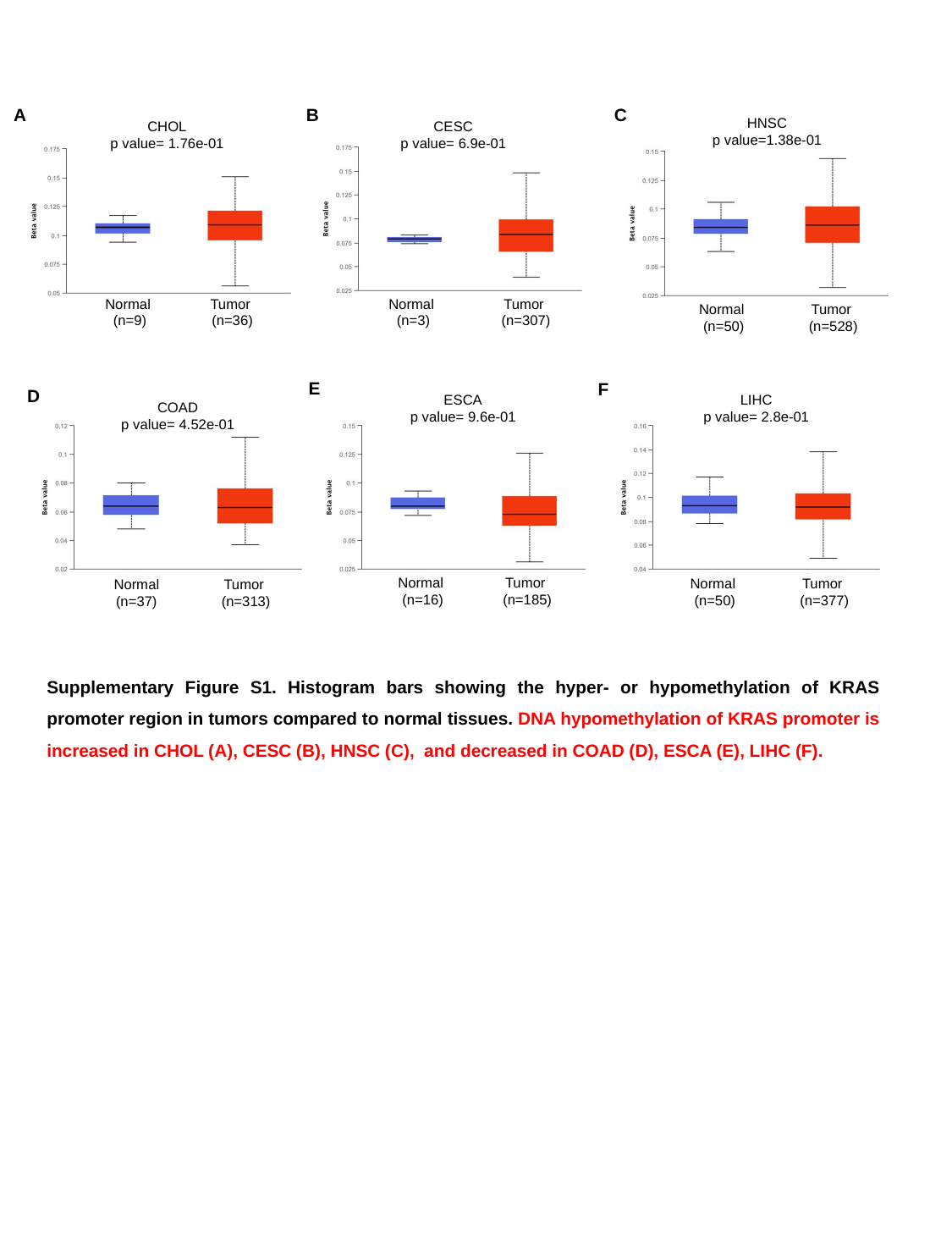

A
CHOL
p value= 1.76e-01
Normal
 (n=9)
Tumor
(n=36)
B
CESC
p value= 6.9e-01
Normal
(n=3)
Tumor
(n=307)
C
HNSC
p value=1.38e-01
Normal
(n=50)
Tumor
(n=528)
E
ESCA
p value= 9.6e-01
Normal
(n=16)
Tumor
(n=185)
F
LIHC
p value= 2.8e-01
Normal
(n=50)
Tumor
(n=377)
D
COAD
p value= 4.52e-01
Normal
(n=37)
Tumor
(n=313)
Supplementary Figure S1. Histogram bars showing the hyper- or hypomethylation of KRAS promoter region in tumors compared to normal tissues. DNA hypomethylation of KRAS promoter is increased in CHOL (A), CESC (B), HNSC (C), and decreased in COAD (D), ESCA (E), LIHC (F).
