# Supplementary material for: Prognostic and Immunotherapeutic Roles of KRAS in Pan-Cancer
Source: Cells. 2022 Apr 22;11(9):1427. doi: 10.3390/cells11091427 (PMC9105487; doi:10.3390/cells11091427)
Supplement: Supplementary file 1 [file cells-11-01427-s001.zip › cells-1666153-supplementary/Supplementary Figure S2.pptx]

## Slide 1
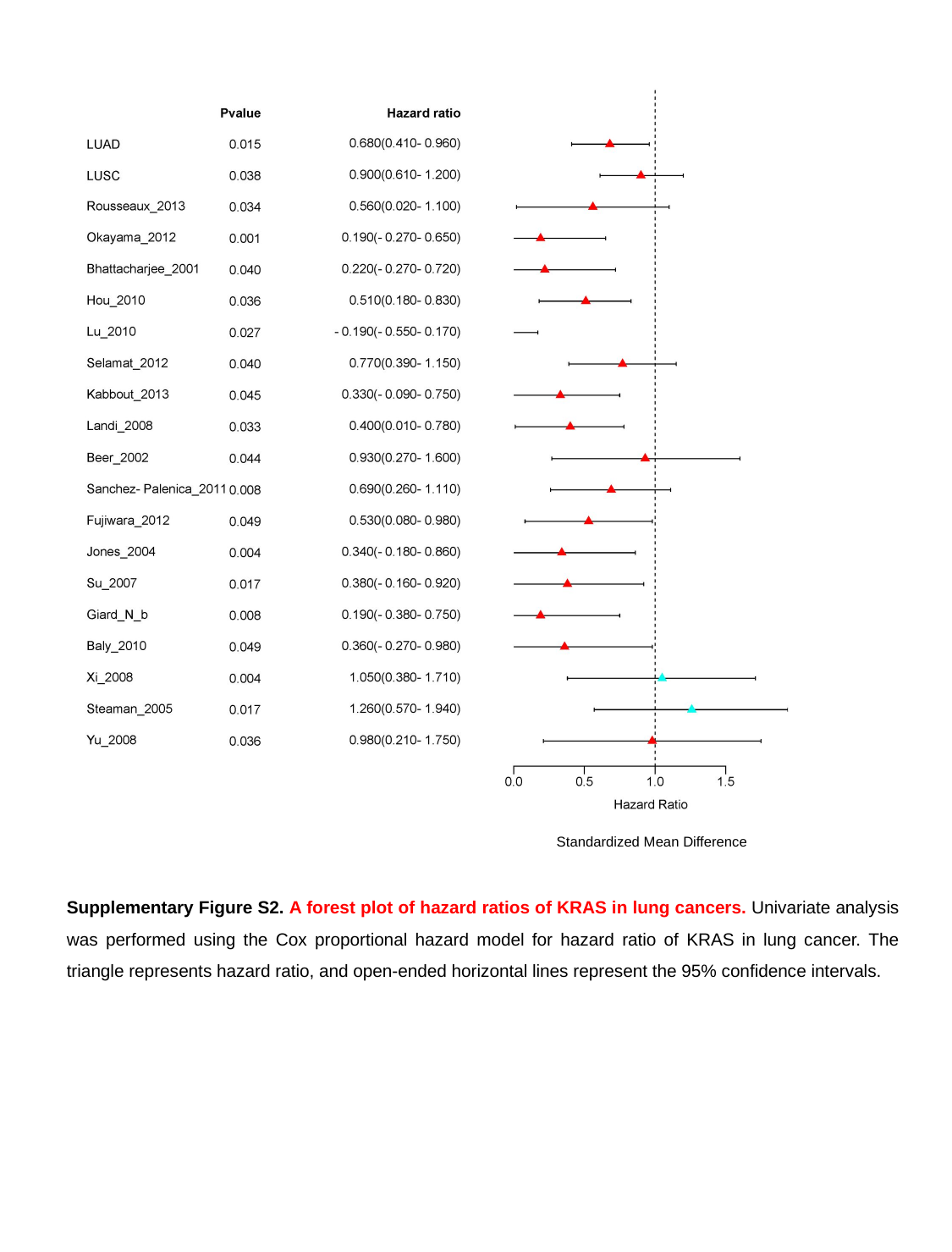

Standardized Mean Difference
Supplementary Figure S2. A forest plot of hazard ratios of KRAS in lung cancers. Univariate analysis was performed using the Cox proportional hazard model for hazard ratio of KRAS in lung cancer. The triangle represents hazard ratio, and open-ended horizontal lines represent the 95% confidence intervals.
