# Supplementary material for: Prognostic and Immunotherapeutic Roles of KRAS in Pan-Cancer
Source: Cells. 2022 Apr 22;11(9):1427. doi: 10.3390/cells11091427 (PMC9105487; doi:10.3390/cells11091427)
Supplement: Supplementary file 1 [file cells-11-01427-s001.zip › cells-1666153-supplementary/Supplementary Figure S3.pptx]

## Slide 1
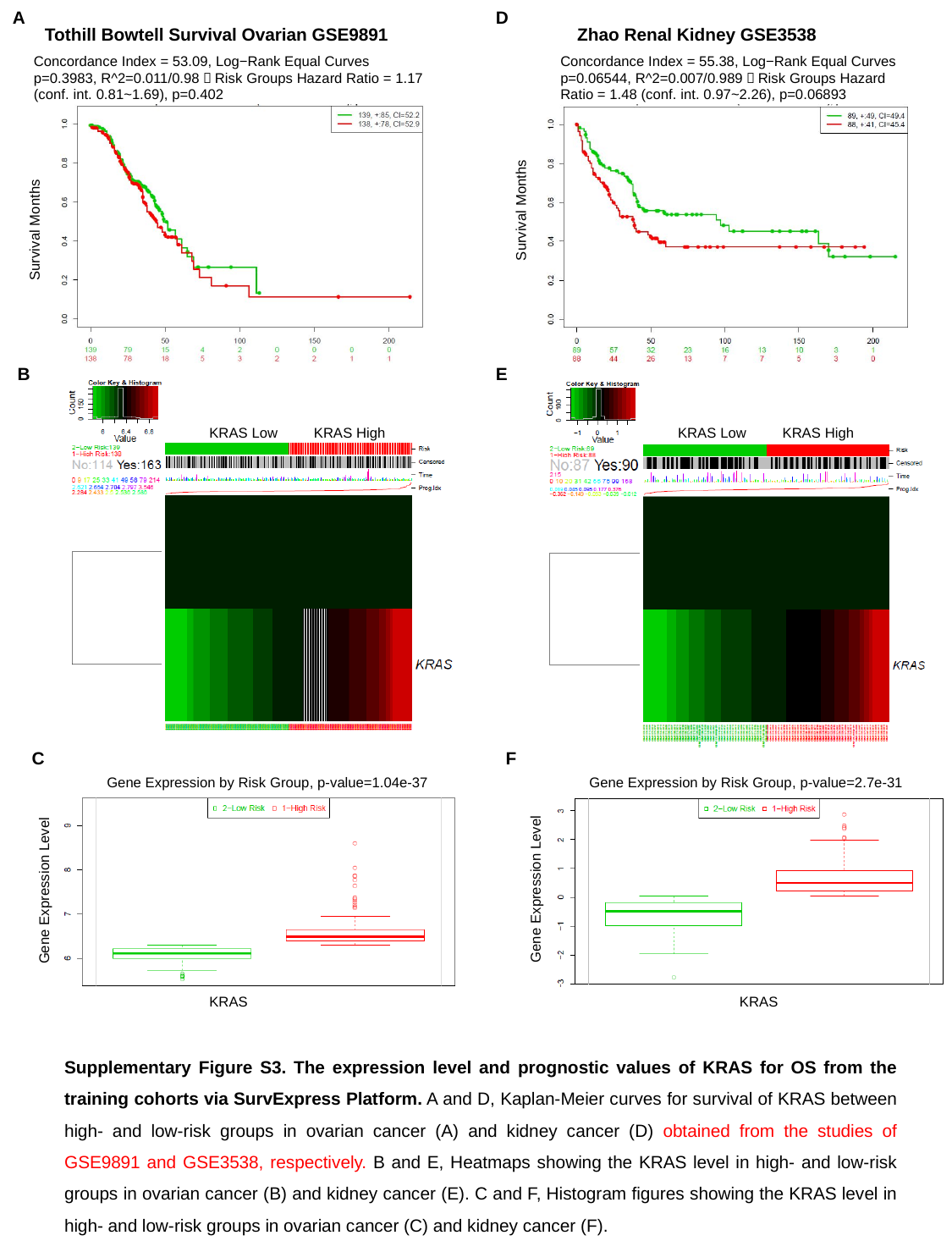

A
D
Tothill Bowtell Survival Ovarian GSE9891
Zhao Renal Kidney GSE3538
Concordance Index = 53.09, Log−Rank Equal Curves p=0.3983, R^2=0.011/0.98，Risk Groups Hazard Ratio = 1.17 (conf. int. 0.81~1.69), p=0.402
Concordance Index = 55.38, Log−Rank Equal Curves p=0.06544, R^2=0.007/0.989，Risk Groups Hazard Ratio = 1.48 (conf. int. 0.97~2.26), p=0.06893
Survival Months
Survival Months
B
E
KRAS Low KRAS High
KRAS Low KRAS High
C
F
Gene Expression by Risk Group, p-value=1.04e-37
Gene Expression Level
KRAS
Gene Expression by Risk Group, p-value=2.7e-31
Gene Expression Level
KRAS
Supplementary Figure S3. The expression level and prognostic values of KRAS for OS from the training cohorts via SurvExpress Platform. A and D, Kaplan-Meier curves for survival of KRAS between high- and low-risk groups in ovarian cancer (A) and kidney cancer (D) obtained from the studies of GSE9891 and GSE3538, respectively. B and E, Heatmaps showing the KRAS level in high- and low-risk groups in ovarian cancer (B) and kidney cancer (E). C and F, Histogram figures showing the KRAS level in high- and low-risk groups in ovarian cancer (C) and kidney cancer (F).
