# Supplementary material for: Prognostic and Immunotherapeutic Roles of KRAS in Pan-Cancer
Source: Cells. 2022 Apr 22;11(9):1427. doi: 10.3390/cells11091427 (PMC9105487; doi:10.3390/cells11091427)
Supplement: Supplementary file 1 [file cells-11-01427-s001.zip › cells-1666153-supplementary/Supplementary Figure S4.pptx]

## Slide 1
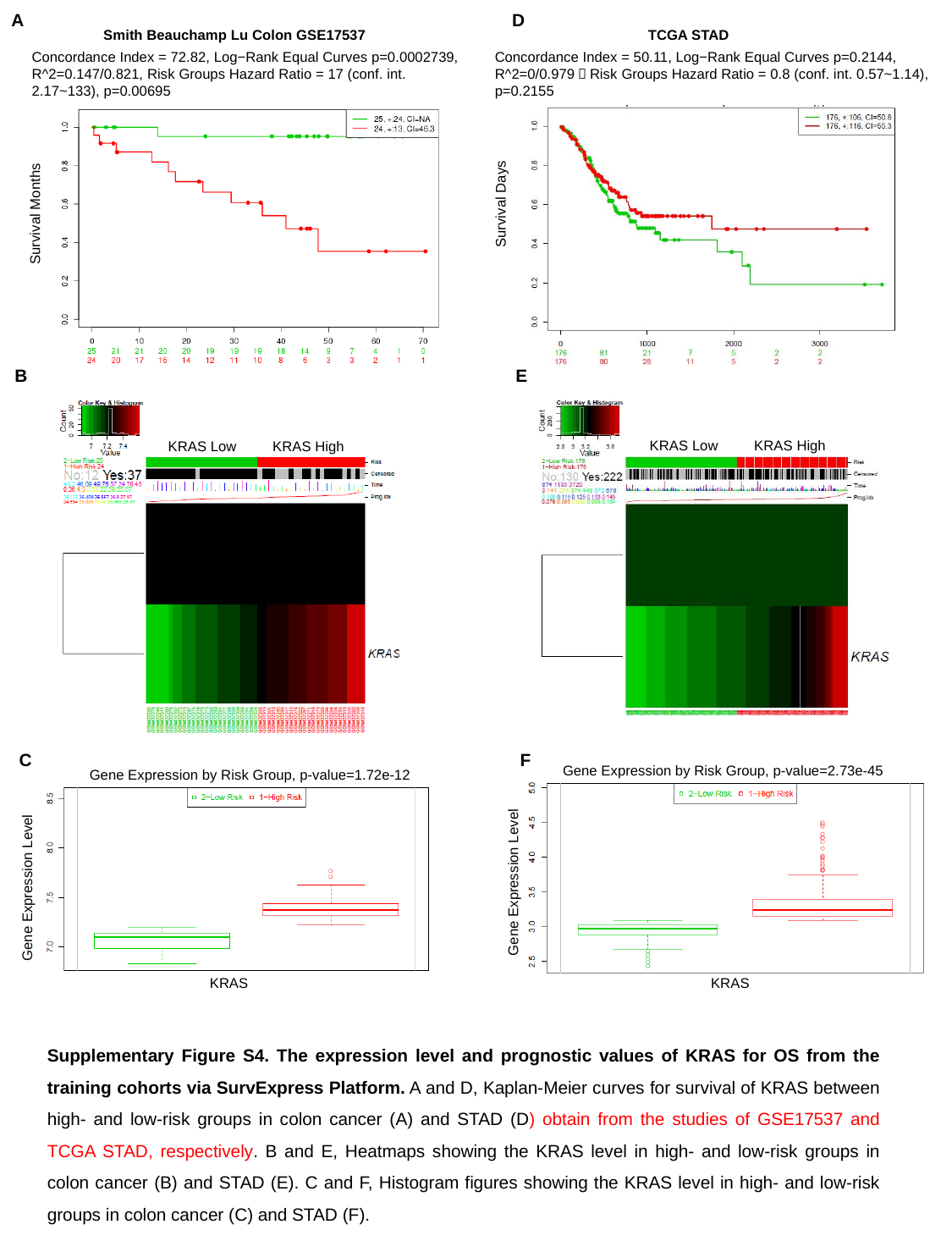

A
Smith Beauchamp Lu Colon GSE17537
Concordance Index = 72.82, Log−Rank Equal Curves p=0.0002739, R^2=0.147/0.821, Risk Groups Hazard Ratio = 17 (conf. int. 2.17~133), p=0.00695
Survival Months
D
TCGA STAD
Concordance Index = 50.11, Log−Rank Equal Curves p=0.2144, R^2=0/0.979，Risk Groups Hazard Ratio = 0.8 (conf. int. 0.57~1.14), p=0.2155
Survival Days
B
E
KRAS Low KRAS High
KRAS Low KRAS High
C
F
Gene Expression by Risk Group, p-value=2.73e-45
Gene Expression Level
KRAS
Gene Expression by Risk Group, p-value=1.72e-12
Gene Expression Level
KRAS
Supplementary Figure S4. The expression level and prognostic values of KRAS for OS from the training cohorts via SurvExpress Platform. A and D, Kaplan-Meier curves for survival of KRAS between high- and low-risk groups in colon cancer (A) and STAD (D) obtain from the studies of GSE17537 and TCGA STAD, respectively. B and E, Heatmaps showing the KRAS level in high- and low-risk groups in colon cancer (B) and STAD (E). C and F, Histogram figures showing the KRAS level in high- and low-risk groups in colon cancer (C) and STAD (F).
