# Supplementary material for: Prognostic and Immunotherapeutic Roles of KRAS in Pan-Cancer
Source: Cells. 2022 Apr 22;11(9):1427. doi: 10.3390/cells11091427 (PMC9105487; doi:10.3390/cells11091427)
Supplement: Supplementary file 1 [file cells-11-01427-s001.zip › cells-1666153-supplementary/Supplementary Figure S5.pptx]

## Slide 1
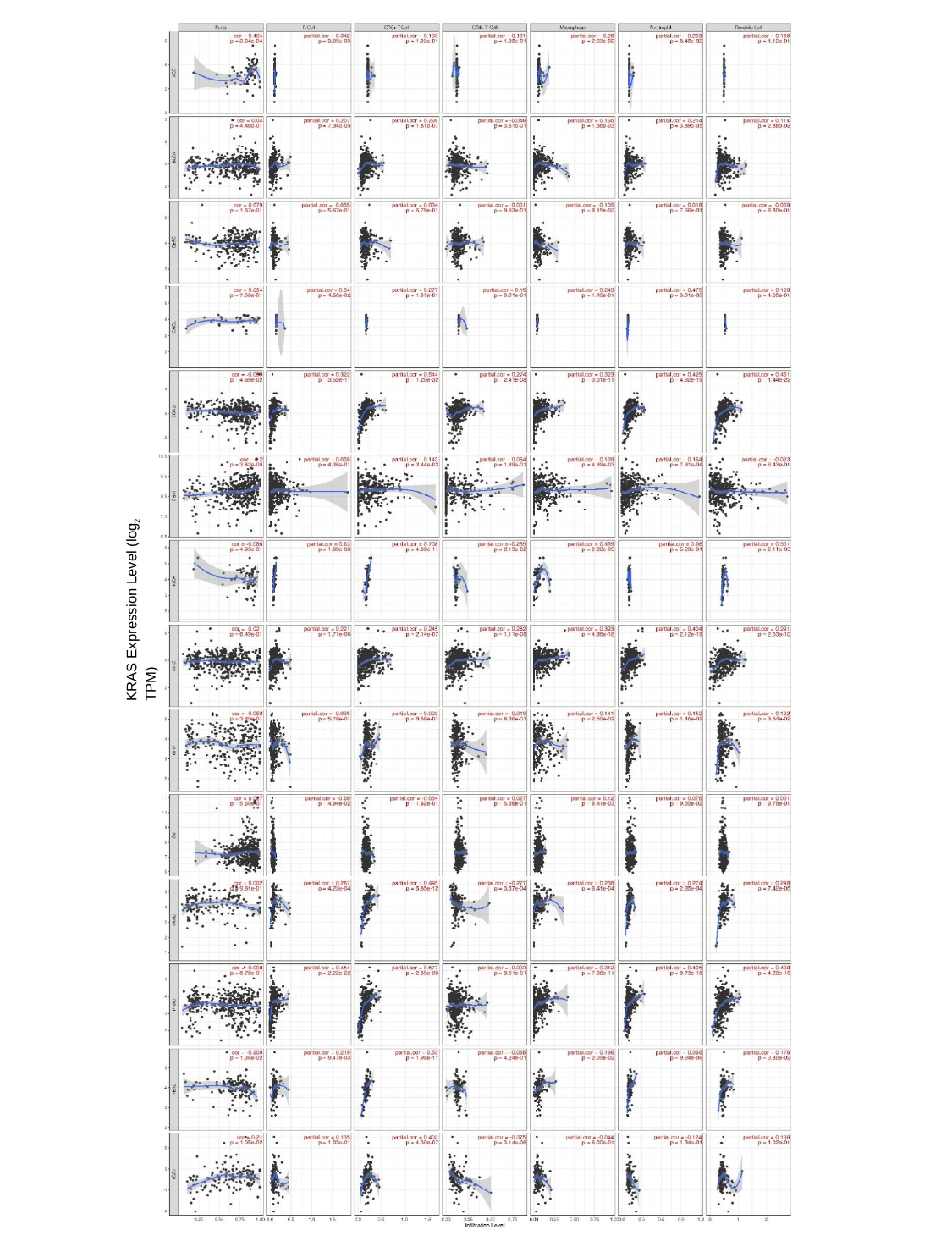

KRAS Expression Level (log2 TPM)

## Slide 2
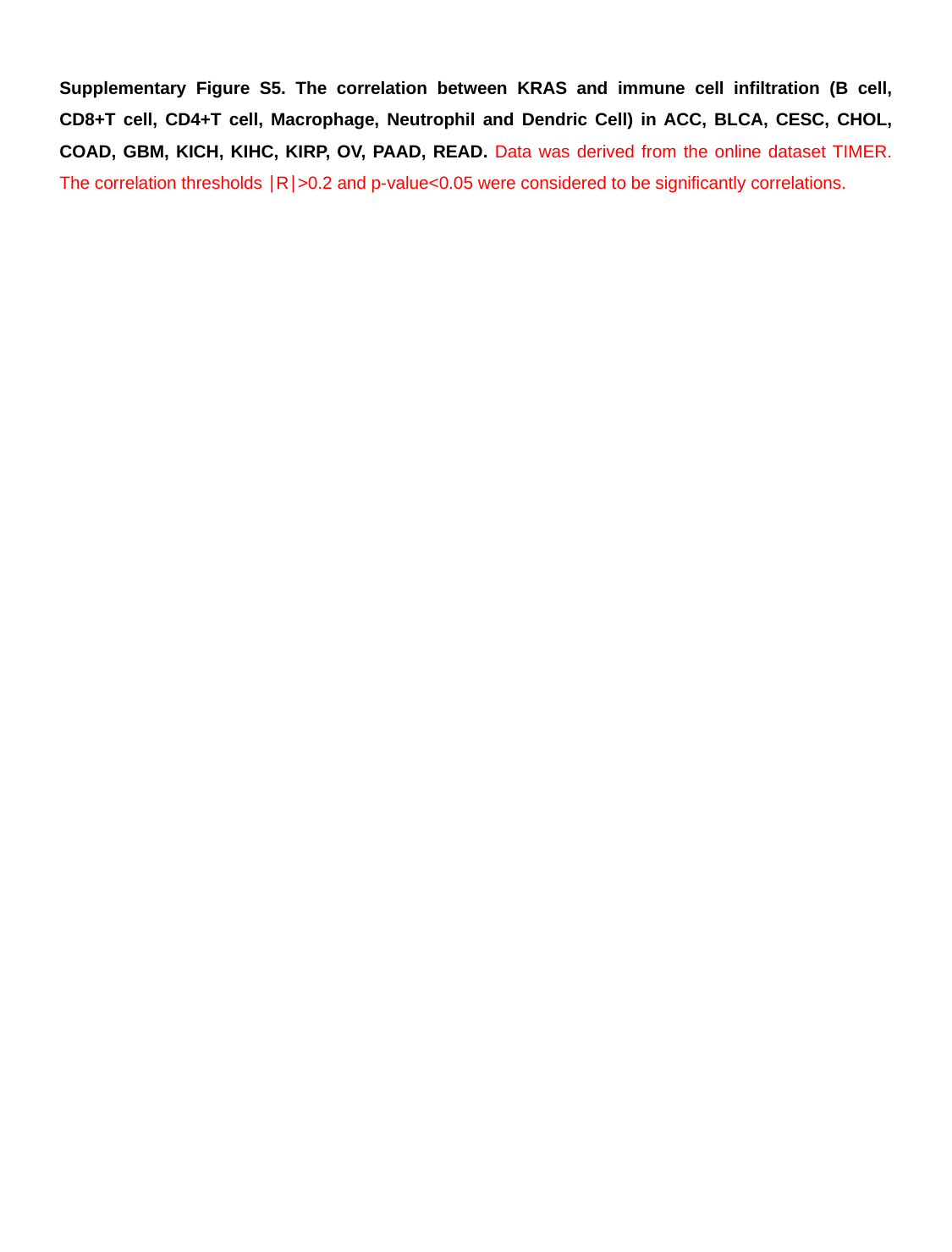

Supplementary Figure S5. The correlation between KRAS and immune cell infiltration (B cell, CD8+T cell, CD4+T cell, Macrophage, Neutrophil and Dendric Cell) in ACC, BLCA, CESC, CHOL, COAD, GBM, KICH, KIHC, KIRP, OV, PAAD, READ. Data was derived from the online dataset TIMER. The correlation thresholds ∣R∣>0.2 and p-value<0.05 were considered to be significantly correlations.
