# Supplementary material for: Prognostic and Immunotherapeutic Roles of KRAS in Pan-Cancer
Source: Cells. 2022 Apr 22;11(9):1427. doi: 10.3390/cells11091427 (PMC9105487; doi:10.3390/cells11091427)
Supplement: Supplementary file 1 [file cells-11-01427-s001.zip › cells-1666153-supplementary/Supplementary Figure S6.pptx]

## Slide 1
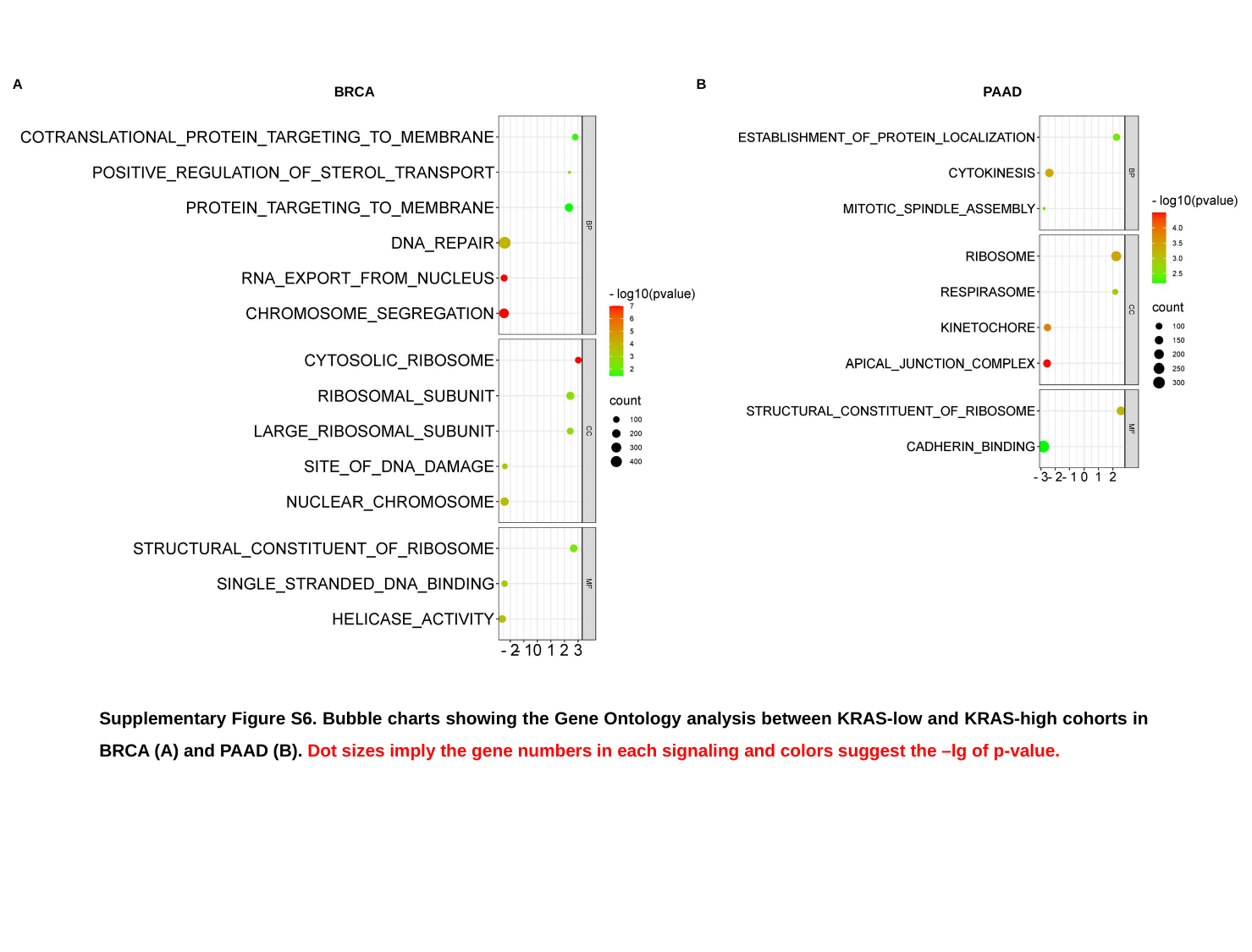

A
BRCA
B
PAAD
Supplementary Figure S6. Bubble charts showing the Gene Ontology analysis between KRAS-low and KRAS-high cohorts in BRCA (A) and PAAD (B). Dot sizes imply the gene numbers in each signaling and colors suggest the –lg of p-value.
